# Evaluating real-time momentary stress and affect in police officers using a smartphone application

**DOI:** 10.1186/s12889-020-09225-z

**Published:** 2020-07-23

**Authors:** Gi Wook Ryu, Yong Sook Yang, Mona Choi

**Affiliations:** Yonsei University College of Nursing, Mo-Im Kim Nursing Research Institute, 50 Yonsei-ro, Seodaemun-gu, Seoul, 03722 Republic of Korea

**Keywords:** Police officer, Ecological momentary assessment, Momentary stress, Affect, Smartphone application

## Abstract

**Background:**

Police officers work under stressful conditions, and the resulting occupational stress may impact their health and well-being through changes in positive affect (PA) and negative affect (NA). It is therefore important to assess officers’ stress, PA, and NA while it is experienced. This study evaluates police officers’ momentary stress and affect in real-world settings using an ecological momentary assessment, and examines the within-person (W) and between-person (B) factors that influence momentary affect.

**Methods:**

Eighty-nine police officers were recruited in South Korea. Participants completed questionnaires about their momentary stress and affect using a smartphone application. The associations between momentary stress, momentary contextual environment, momentary PA, and momentary NA were examined using mixed modeling.

**Results:**

Social overload (W: -.37), work discontent (W: −.45, B: −.73), social tension (W: −.79, B: −.67), and pressure to perform (W: −.29, B: −.49) were significantly associated with lower PA. Work overload (B: .33) and social isolation(W: .48, B: .31) were significantly associated with higher PA. Being with family (W: .71, B: .91) and friends (W: 1.89, B: 2.45) were significantly associated with higher PA. Being at home or other places away from the work place were significantly associated with higher PA (W: 1.01) and when patrolling or investigating were associated with lower PA (B: − 1.13). Lack of social recognition (W: 1.74, B: 2.33), work discontent (W: 1.59, B: 1.88), social tension (W: 1.74, B: 2.92), and pressure to perform (W: .78, B: 1.92) were significantly associated with higher NA. Being with colleagues (W: − 1.43), family (W: -1.38, B: − 2.66) and friends (W: -1.78, B: − 2.45) were associated with lower NA.

**Conclusions:**

Momentary within-person and between-person stress factors and contextual factors influenced police officers’ momentary affect. These factors should be considered when developing interventions to mitigate stress and improve affect in police officers.

## Background

Occupational stress is a well-known condition associated with high levels of distress, depression, anxiety, and burnout among police officers [1]. In the course of their daily work, police officers deal with various crimes—including domestic violence, physical assaults, and sexual assaults—and those who perpetrate them, which leads to unpredictable circumstances. They are exposed to numerous potentially traumatic and critical situations in their work [2], and their daily activities involve public contact that requires emotional labor [3]. They are also exposed to organizational stressors, such as bureaucratic procedures, record keeping, and human resource management [4, 5].

Police officers’ work environment and high occupational stress can have negative effects on their health. For example, high stress and changes in circadian rhythms due to shift work can be risk factors for cardiovascular diseases [6]. For example, a previous study showed the hazard ratios for angina pectoris and cerebrovascular disease in police officers to be 1.52 and 1.36, respectively, significantly higher than those of other government workers [7]. In a study conducted in the United States, the life expectancy of police officers was shown to be 21.9 years lesser than that of the general population [8]. Occupational stress has also been shown to be positively correlated with anxiety, depression, and post-traumatic stress disorder [9–11]. Therefore, police officers’ stress and affect can negatively influence their health, increase absenteeism, and decrease productivity, ultimately having a negative impact on the general public and national security [12].

Stress is a psychological state involving the relationship between a person and their environment that overwhelms their resources and threatens their well-being [13]. According to Lazarus and Folkman’s transitional theory, people evaluate the meaning of what is happening to them through cognition [13]. Cognitive outcomes can have both immediate and long-term effects. Immediate effects include positive affect (PA), negative affect (NA), physiological changes, and encounter quality, while long-term effects include somatic health, well-being, and social function [13].

PA and NA refer to an individual’s consciously accessible feelings and subjective experience [14, 15]. PA is characterized by enthusiasm, confidence, and energy, while NA is characterized by factors such as guilt, fear, and nervousness [16]. PA can influence individual well-being, which is associated with improved work performance, relationship satisfaction, and improved health [17].

Systematic reviews of existing studies on psychological outcomes for police officers revealed that factors associated with stress include higher job demands, poor relationships with colleagues, job pressure, low rewards, role ambiguity, and lack of social support [3, 18]. Contextual factors were shown to include traumatic events, injuries while on duty, direct threats to one’s life, taking a life in the line of duty, and frequent exposure to crime [3, 14]. These factors influence officers’ affect, causing feelings such as anger, anxiety, and guilt [19]. However, previous studies have been limited to between-person assessments, creating an inability to examine time-varying within-person factors that impact stress, as well as the momentary effect of stress on affect change, according to personal attributes and environment [13, 20]. Therefore, both within-person and between-person stress and affect should be evaluated within this population.

In this context, an ecological momentary assessment (EMA) is a useful approach. EMA collects real-time data on the behaviors and experiences of participants by describing phenomena or participant characteristics [21]. Methodologically, EMA is a longitudinal method that repeatedly measures participants’ time-dependent variables through self-reports made in a natural environment. EMA has previously been used in medical and psychological research to examine changes in behavior, stress, and affect [21, 22]. Furthermore, recent developments in information technology have made it possible to survey participants and obtain information on psychological properties using devices such as smartphones, personal digital assistants, and sensors [23]. A substantial advantage of EMA is that dynamic changes in participants’ behaviors and feelings in real-world settings can be recorded with reduced recall bias [22, 24]. Moreover, it captures the interaction between participants and their contextual environment by examining and comparing within- and between-person factors [22]. A study that applies EMA can provide more accurate and diverse information to health experts and administrators, compared with cross-sectional studies. Thus, exploring the contextual and stress factors of within- and between-person affect changes in a real-world setting will help contribute towards mitigating stress and NA in police officers.

This study aimed to examine the associations between momentary within- and between-person affect (PA, NA), stress factors, and context by using EMA via a mobile device application. The following hypotheses were investigated: overall momentary stress correlates with momentary PA and NA, and overall momentary stress predicts momentary PA and NA (Hypothesis 1); and within- and between-person momentary PA and NA are influenced by stress factors and contextual information, such as with whom, location, and task type (Hypothesis 2).

## Methods

### Participants

Using convenience sampling, 97 police officers in the metropolitan area of Gyeonggi Province, South Korea, were recruited between July and September 2018. The inclusion criteria were police officers who were currently working at a police station, used Android smartphones, and were able to respond to surveys using the application. As the developed mobile application was only available for Android phones [25], those who used smartphones with other operating systems were excluded. All participants provided informed written consent before participating in the study. The study was conducted in accordance with the Declaration of Helsinki, and the protocol was approved by the Ethics Committee of the Yonsei University Health System (approval number: Y-2018-0035).

### Measurements

The measurements included baseline variables such as demographic characteristics, trait affect, and occupational stress. Momentary measures were stress, affect, and contextual information. The study variables at baseline and momentary measures are summarized in Table 1.

**Table 1:** Study variables at all timepoints

Variables	Baseline	EMA assessment						
		Day 1	Day 2	Day 3	Day 4	Day 5	Day 6	Day 7
Demographic characteristics	○							
Occupational stress (KOSS-SF)	○							
Trait affect (K-PANAS)	○							
Momentary stress (TICS)		○	○	○	○	○	○	○
Momentary affect (PsyMate)		○	○	○	○	○	○	○
Contextual information		○	○	○	○	○	○	○

*EMA* Ecological Momentary Assessment; *KOSS-SF* Korean Occupational Stress Scale-Short Form; *K-PANAS* Korean Positive Affect and Negative Affect Schedule, *TICS* Trier Inventory for Chronic Stress

### Baseline self-report measures

Data on participants’ occupational stress and affect were obtained, along with demographic information including age, sex, marital status, department of work, rank, working duration, and shift hours. We used the Korean Occupational Stress Scale-Short Form (KOSS-SF) to evaluate participants’ occupational stress [26]. This 24-item instrument comprises seven categories related to work stress: work demands, insufficient work control, interpersonal conflict, work security, organizational system, lack of reward, and occupational climate. Each item is rated on a 4-point Likert scale ranging from (1) “not at all” to (4) “extremely.” The calculation method of the scores for each category is ([acquired score – number of items] / [highest possible score – number of items]) * 100, which was converted to a total of 100 points. The total score for job stress is the average of the sum of the scores of each category and ranges from 0 to 100, with higher scores indicating higher stress. The Cronbach’s alpha of this scale in the present study was .767.

To assess affect, the Korean version of the Positive Affect and Negative Affect Schedule (PANAS) was used [27, 28]. This instrument comprises 10 items each on PA and NA. PA refers to a state of high energy, complete concentration, and pleasurable engagement, while NA refers to a state of subjective distress and unpleasurable engagement. Each item is rated on a 5-point Likert scale ranging from (0) “not at all” to (4) “extremely.” The total score ranges from 0 to 40 each for PA and NA, with higher scores indicating higher affect. The Cronbach’s alphas for the PA and NA scales in the present study were .945 and .933, respectively.

### Ecological momentary assessment measures

#### Momentary stress

To assess real-time momentary stress, we used the Trier Inventory for Chronic Stress (TICS), adapted from the German version [29, 30]. Prior to this study, we received permission from the developers to translate and use the TICS. The scale was translated into Korean using the translation and back-translation method. Based on the original TICS [29, 30], our scale uses an eight-item construct with the following items: work overload (“I did a lot of work”), social overload (“I dealt a lot with other people’s matters”), pressure to perform (“I performed some of my tasks inadequately”), work discontent (“Others undervalued my work”), excessive demands at work (“I felt discontented with the type of work I am doing”), lack of social recognition (“I had a disagreement with someone”), social tension (“I performed tasks that allowed no mistakes”), and social isolation (“It was important to ensure good relations with another person”). Each item is rated on a 5-point Likert scale ranging from (1) “not at all” to (5) “extremely.” The total score ranges from 8 to 40, with higher scores indicating higher momentary stress. The Cronbach’s alpha of the scale in the present study was .839.

#### Momentary affect

To evaluate real-time momentary affect, the PsyMate survey was used [31]. We received permission from the authors to translate and use the PsyMate, and translated it into Korean using the translation and back-translation method. The survey comprises 13 items, of which four items evaluate PA and nine items evaluate NA. An example of momentary PA items is “I feel cheerful, satisfied, relaxed, and am generally feeling well.” An example of momentary NA items is “I feel lonely, guilty, worried, down, threatened, insecure, irritated, frightened, and suspicious.” Each item is rated on a 7-point Likert scale ranging from (1) “not at all” to (7) “extremely.” The total score ranges from 4 to 28 for PA and 9 to 63 for NA, with higher scores indicating higher momentary affect. The Cronbach’s alphas of the scale in the present study were .946 for PA and .945 for NA.

#### Momentary contextual information

We collected momentary contextual information from the participants, which included who they were with, where they were, and what they were doing. These items provided information concerning the context of a participant’s experience of momentary stress and affect. The choices for each item were structured based on the work guidelines for Korean police officers, which were reviewed by experts. For the “with whom” item, the choices were alone, colleagues, boss, civilians, family, or friends. For the location-related item, the choices were police station, working outside, home, or other. For the task-related item, participants were distinguished based on their type of work, such as administration, patrolling or investigating, resting, or other.

### Procedure

Prior to data collection, we explained the research to the captains of each police station, and they provided us with permission to conduct the research. The research team provided a booklet with instructions on using the mobile application, and the application was installed on all participants’ smartphones. Participants completed the initial questionnaires and EMA items using their smartphones. The EMA application was developed by the research team, and the details are described in a previously published article [25]. The application consists of questionnaires on participants’ stress, affect, and momentary contextual information.

The EMA was performed over seven consecutive days; real-time self-reported responses were prompted by the application’s alarm function. Surveys were conducted four times a day from 8:00 to 9:00, 13:00 to 14:00, 17:00 to 18:00, and 21:00 to 22:00. We determined the number and timing of alarms based on the EMA methodological systematic review [22]. It should be noted that the alarm intervals were not constant, because we considered the participants’ commute and lunch/dinner times. The alarm was set at semi-random intervals within 60-min periods. Participants received gift coupons as an incentive for their participation. We contacted participants who missed all four responses on the first day of EMA data collection to help solve any problems impeding participation, such as technical difficulties or intention to drop out. During the study, participants were encouraged to contact the research team via telephone call or text message to address any questions.

### Statistical analysis

Data were analyzed using SPSS version 23 and STATA version 13. We included data from participants who completed the survey three times or more, as this is considered to be suitable for a longitudinal analysis, based on the work of Hoffman [20]. Participants’ baseline and momentary stress and affect were analyzed using descriptive statistics. To assess overall momentary stress levels, total scores were used. To analyze the influence of momentary stress on momentary affect with mixed modeling, each stress factor was applied based on the momentary stress questionnaire (TICS) construct to determine the influence of each stress construct.

To test Hypothesis 1, Pearson’s correlation coefficients were used to examine correlations between overall momentary stress and momentary PA and NA. Additionally, a simple linear regression analysis was performed to predict momentary affect by overall momentary stress. To test Hypothesis 2, Momentary PA and NA related to momentary stress were examined on two levels—within-person (level 1) and between-person (level 2)—using mixed modeling for a longitudinal analysis method [20]. Data from EMA studies are inherently multilevel with (level 1) a specific individual’s variation nested within (level 2) individuals. Thus, mixed-effects models investigated the disaggregated effects of within-person changes and between-person differences in momentary PA and NA related to momentary stress.

Factors predicting momentary affect were analyzed separately using three models—stress factors, context, and trait variables—at levels 1 and 2. In Model 1, real-time predictors with stress factors were included as fixed effects. In Model 2, we added contextual factors, such as with whom and location, and task-related factors to Model 1. In Model 3, we added trait variables such as age, sex, rank, work duration, occupational stress, and baseline affect to Model 2. As traits, baseline occupational stress and affect can influence momentary affect [32]; thus, baseline stress and affect were included in the adjusted model. Furthermore, momentary affect and stress can change owing to an event and vary with time, surrounding context, and inter-individual differences [20]. Table 2 shows the variables included in each model and level; therefore, these models are three sets of models at level 1 and level 2.

**Table 2:** Levels, models, and variables related to momentary positive and negative affect

Level	Model	Variable
1. Within-person	1. Stress factors	work overload, social overload, pressure to perform, work discontent, excessive demands at work, lack of social recognition, social tension, and social isolation
	2. Context	+ with whom, location-related, and task-related factors
	3. Trait variables	+ age, sex, rank, work duration, occupational stress, and baseline affect
2. Between-person	1. Stress factors	work overload, social overload, pressure to perform, work discontent, excessive demands at work, lack of social recognition, social tension, and social isolation
	2. Context	+ with whom, location-related, and task-related factors
	3. Trait variables	+ age, sex, rank, work duration, occupational stress, and baseline affect

*PA* Positive affect; *NA* Negative affect

## Results

### Participant characteristics

We initially recruited 112 participants, 15 of whom (13.4%) did not respond to our EMA survey; thus, a total of 97 police officers participated in this study. After data were excluded from participants who completed the momentary survey fewer than three times (*n* = 8), data from 89 participants were included in the final analyses, which comprised 1613 of 2492 entries (Completion rate: 64.7%, SD: 22.69, Range: 10.71–100%). The average number of answered prompts per participant was 17, and the range was 3 to 28 times per participant. The median response time for completion was 38 min.

The mean age of the participants was 37.15 (± 10.52) years. There was a higher proportion of male participants (*n* = 84; 94.4%) and more than half were married (*n* = 50; 56.2%). Regarding their rank, police officers were the most common (*n* = 36; 40.4%), followed by inspectors or higher (*n* = 30; 33.7%), then senior police officers or assistance inspectors (*n* = 23; 25.8%). The most common work department was the public safety division (*n* = 58; 65.2%). The mean years of work experience was 10.42 years (± 10.09), and 64 participants (71.9%) performed shift work (Table 3).

**Table 3:** General and work-related participant characteristics (*n* = 89)

Variables	Sub-category	Mean (± SD) or *n* (%)
Age (years)		37.15 (± 10.52)
Sex	Male	84 (94.4)
	Female	5 (5.6)
Marital status	Single	39 (43.8)
	Married	50 (56.2)
Rank	Police officer	36 (40.4)
	Senior police officer/Assistance inspector	23 (25.8)
	Inspector or higher	30 (33.7)
Department of work	Public safety department	58 (65.2)
	Detective division	3 (3.4)
	Traffic affairs division/ Riot police corps	23 (25.8)
	Command center/ Women & juvenile affairs division	5 (5.6)
Working duration (years)		10.42 (± 10.09)
Shift working	Yes	64 (71.9)
	No	26 (28.1)

### Baseline and momentary stress and affect

The results of the baseline investigation showed a mean occupational stress score of 43.01 (± 8.67) out of 100, while mean PA and NA scores were 17.12 (± 7.23) and 8.00 (± 7.50) out of 40, respectively. The mean overall momentary stress score was 16.43 (±4.32) out of 40. The mean momentary PA score was 18.24 (±3.37) out of 28, and the mean momentary NA score was 19.59 (±8.44) out of 63. The intraclass correlation coefficient (ICC) scores were 0.60, 0.45, and 0.66 for momentary stress, momentary PA, and momentary NA, respectively. This means that the ICC scores for momentary stress showed a 60% variation; there was a 45% variation in momentary PA and a 66% variation in momentary NA (Table 4).

**Table 4:** Means, standard deviations, and intraclass correlation coefficients (ICC) at EMA measurement and baseline (*n* = 89)

Variables (Range)	Mean	SD	ICC
Overall momentary stress (8–40)	16.43	4.32	0.60
Momentary PA (4–28)	18.24	3.37	0.45
Momentary NA (9–63)	19.59	8.44	0.66
Occupational stress (0–100)	43.01	8.67	
PA (0–40)	17.12	7.23	
NA (0–40)	8.00	7.50	

*PA* Positive affect; *NA* Negative affect

Overall momentary stress, momentary PA, and momentary NA were significantly correlated (*p* < .01). Overall momentary stress was positively correlated with occupational stress and NA. Momentary PA was negatively correlated with occupational stress and positively correlated with PA. Momentary NA was positively correlated with NA (Table 5).

**Table 5:** Correlations among overall momentary stress, momentary affect, baseline occupational stress, and baseline affect (*n* = 89)

Variables	Overall momentary stress	Momentary PA	Momentary NA	Baseline occupational stress	Baseline PA	Baseline NA
	*r* (*p*)					
Overall momentary stress	1	-.325 (.002)	.758 (< .001)	.213 (.045)	-.016 (.881)	.309 (.003)
Momentary PA		1	-.498 (< .001)	-.276 (.009)	.330 (.002)	-.048 (.653)
Momentary NA			1	.182 (*.*079)	-.063 (.517)	.417 (.001)
Baseline occupational stress				1	-.202 (.058)	.110 (.001)
Baseline PA					1	.353 (.001)
Baseline NA						1

*PA* positive affect, *NA* negative affect

**Fig. 1:**
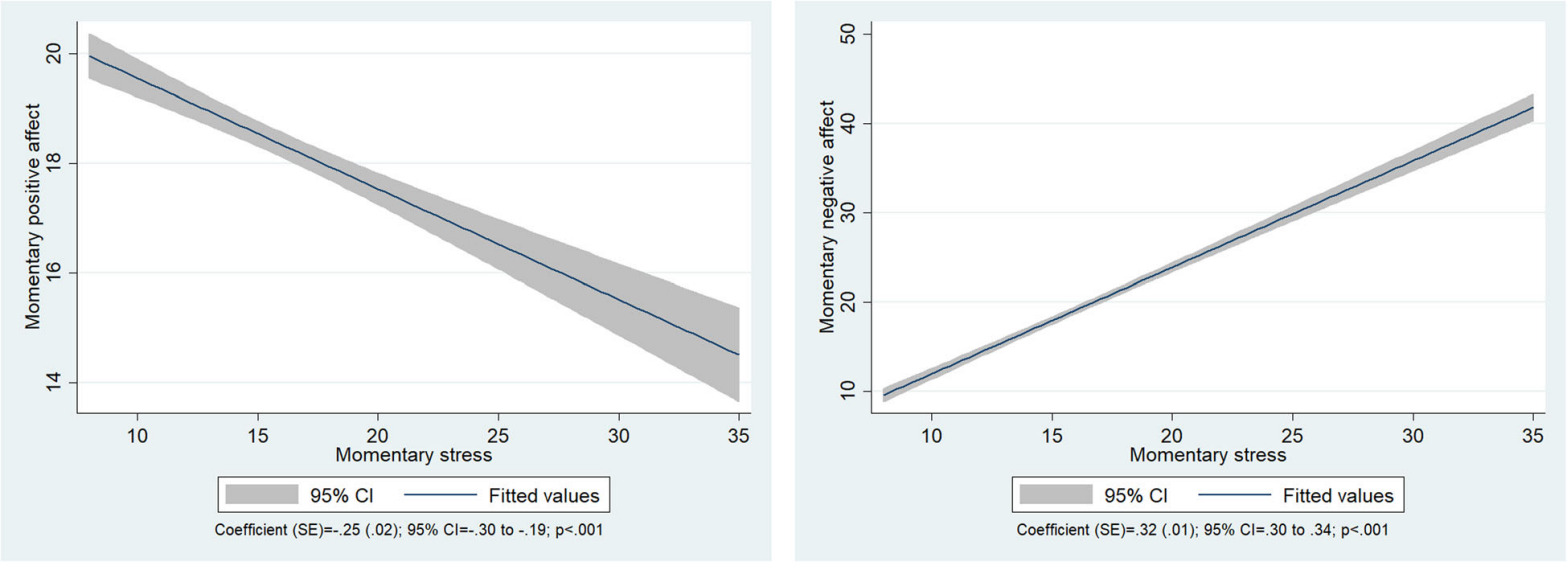
Comparison of the results of overall momentary stress predicting momentary PA and NA (*n* = 1613)

Overall momentary stress predicted momentary PA and NA. The coefficient value of momentary PA by momentary stress was statistically significant (coefficient = −.25; *p* < .001). The coefficient value of momentary NA by momentary stress was also significant (coefficient = .32; *p* < .001; Fig. 1).

### Within- and between-person predictors of momentary PA and NA

The results showed that momentary PA and NA were influenced by the source of stress (work overload, social overload, pressure to perform, work discontent, excessive demands at work, lack of social recognition, social tension, and social isolation) and context (with whom, location-related, and task-related factors) on a within-person level (level 1) and a between-person level (level 2). Table 6 shows the parameter estimates from the mixed effect model for within-person (level l) and between-person (level 2) PA/NA of Model 3 that were analyzed for momentary PA and NA predictions and included stress factors, contextual factors, and traits for adjustment.

**Table 6:** Model 3: Fixed-effect parameter estimates of within-person and between-person stress factors of momentary PA and NA based on multivariate mixed modeling (*n* = 1613)

Variables	Momentary PA				Momentary NA			
	Within-person (Level 1)		Between-person (Level 2)		Within-person (Level 1)		Between-person (Level 2)	
	Coef. (SE)	*p*	Coef. (SE)	*p*	Coef. (SE)	*p*	Coef. (SE)	*p*
Stress factors (by TICS)								
Work overload	.25 (.14)	.064	.33 (.16)	.037	.04 (.21)	.866	−.35 (.26)	.177
Social overload	−.37 (.14)	.011	−.20 (.16)	.208	.09 (.22)	.703	−.13 (.26)	.632
Excessive demands at work	−.21 (.15)	.172	−.11 (.18)	.523	.37 (.24)	.117	.32 (.29)	.272
Lack of social recognition	−.12 (.20)	.538	.23 (.23)	.316	1.74 (.31)	< .001	2.33 (.37)	< .001
Work discontent	−.45 (.14)	.002	−.73 (.16)	< .001	1.59 (.22)	< .001	1.88 (.26)	< .001
Social tension	−.79 (.16)	< .001	−.67 (.18)	< .001	1.74 (.25)	< .001	2.92 (.29)	< .001
Pressure to perform	−.29 (.14)	.039	−.49 (.14)	< .001	.78 (.22)	< .001	1.92 (.22)	< .001
Social isolation	.48 (.10)	< .001	.31 (.10)	.002	.10 (.15)	.519	.24 (.16)	.138
Context With whom (ref. being alone)								
Colleagues	.62 (.42)	.142	.49 (.51)	.347	−1.43 (.66)	.029	−.49 (.83)	.556
Boss	.22 (.46)	.636	−.53 (.56)	.344	−.58 (.72)	.424	−.12 (.91)	.896
Civilians	.69 (1.22)	.576	1.34 (1.48)	.362	1.50 (1.89)	.429	1.94 (2.41)	.423
Family	.71 (.24)	.003	.92 (.27)	.001	−1.38 (.37)	< .001	−2.66 (.44)	< .001
Friends	1.89 (.40)	< .001	2.45 (.47)	< .001	−1.78 (.63)	.004	−2.45 (.77)	.001
Location (ref. police station)								
Working outside	−.14 (.34)	.684	−.40 (.40)	.316	−.47 (.52)	.365	.09 (.65)	.892
Home or other	1.01 (.51)	.048	.86 (.61)	.160	−1.18 (.79)	.134	− 1.36 (1.00)	.172
Type of task (ref. administration)								
Patrolling or investigating	−.54 (.42)	.202	−1.13 (.49)	.020	−.21 (.66)	.753	-.64 (.80)	.414
Resting or other	.00 (.60)	.998	−1.17 (.71)	.100	−.73 (.93)	.432	1.02 (1.16)	.378

These multivariate mixed model analyses for Model 3 were performed for momentary PA and NA as dependent variables and stress factors, contextual factors, and traits as independent variables at within-person (level l) and between-person (level 2) levels

*PA* Positive affect; *NA* Negative affect; *TICS* Trier Inventory for Chronic Stress

NOTE: age, sex, rank, work duration, occupational stress, and baseline affect were adjusted

The questionnaire items and construct follow that of TICS [29, 30]; we have attached the questionnaire as a supplement (S1).

At the within-person level (level 1), social isolation was positively associated with momentary PA. Social overload, work discontent, social tension, and pressure to perform were negatively associated with momentary PA. Regarding contextual factors, momentary PA increased when participants were with family and friends and when they were at home or other places away from the workplace. At the between-person level (level 2), work overload and social isolation were positively associated with momentary PA. Work discontent, social tension, and pressure to perform were negatively associated with momentary PA. Regarding contextual factors, momentary PA increased when participants were with family and friends and decreased when they were patrolling or investigating.

At the within-person level (level 1), lack of social recognition, work discontent, social tension, and pressure to perform were positively associated with momentary NA. Among contextual factors, momentary NA decreased when participants were with colleagues, family, and friends. At the between-person level (level 2), lack of social recognition, work discontent, social tension, and pressure to perform were positively associated with momentary NA. Among contextual factors, momentary NA decreased when participants were with family and friends.

## Discussion

This study used an EMA method to examine the within- and between-person differences in momentary stress factors and contextual information on momentary affect experienced by police officers. The results showed that the participants’ contextual environment in daily life influenced their momentary stress and affect on both within- and between-person levels.

Hypothesis 1—positing that overall momentary stress is correlated with momentary PA and NA and overall momentary stress predicts momentary affect—was supported. Lazarus and Folkman reported that an individual’s stress level is determined by their cognitive appraisal, and that appraisal and re-appraisal occur continuously, based on changes in the relationship between the individual and the environment [13]. Based on this theory, cognitive appraisal of a momentary real-life contextual environment would influence momentary stress. Furthermore, the outcome of a cognitive appraisal and response may influence momentary affect, which was one of the final outcomes of this study. Furthermore, considering the slope that predicts momentary affect, the confidence interval range of the momentary NA slope was slightly steeper than that of momentary PA. This indicates that momentary NA was more sensitive to momentary stress than momentary PA, in terms of response. This can be explained by the condition of neuroticism, an individual’s tendency to experience NA in response to stressful situations [33]. Neuroticism may result in difficulties adapting and coping with a stressful environment [33, 34].

Hypothesis 2 was partially supported. Work discontent, social tension, and pressure to perform influenced momentary affect at the within- and between-person levels. This finding is consistent with those of previous studies that report job demands, job pressure, high effort, and high workload are stress factors [2, 18]. Individuals in emergency situations—such as police officers—often experience a lack of control, psychological pressure, and distress [2]. These work-related burdens may worsen their psychological stress and social tension, because they have interpersonal relationships with many people, including civilians. This study showed that participants experienced psychological pressure and distress due to work-related factors, which led to decreased PA and increased NA, at both the within-person and between-person levels.

There were some differences in the factors that significantly influenced within- and between-person momentary PA. Work overload and patrolling or investigating influenced between-person, but not within-person, momentary PA. This finding was similar to that of a previous study, which showed that outdoor patrol officers experienced more stress and anxiety than officers who worked indoors [4]. This may be because these officers have difficulties in controlling the outdoor environment. In the case of investigating, police officers have to deal with different types of victims, and they exposed to emotional trauma or externally inflicted victim trauma [35].

Being with family and friends increased momentary PA and decreased NA, at both the within- and between-person levels. Moreover, being with colleagues decreased within-person momentary NA. This indicates that social support from family, friends, and colleagues is important for police officers who experience social isolation and rejection because of the characteristics of their work [36]. Social support can play a buffering role when officers experience stress or traumatic events [37]. Further, social support through interpersonal relationships mitigates psychological symptoms. Therefore, developing social support strategies is essential to mitigate stress and NA.

To our knowledge, this study is the first to investigate the effects of momentary stress and contextual factors on real-time momentary PA and NA in police officers. The findings indicate that within- and between-person momentary affect (both PA and NA) were influenced and changed by momentary stress factors and contextual factors. These factors can be considered when designing interventions to improve PA and reduce NA in police officers. For example, previous studies showed the effectiveness of interventions, such as mindfulness or cognitive change techniques, that were provided to help change police officers’ perceptions of stress and contextual factors [38, 39]. Additionally, as momentary PA and NA are influenced by task type—for example, patrolling or investigating—it is necessary to implement individual support programs that can assist police officers in managing the negative impact to stress and affect caused by a particular task.

As being with family and friends was shown to decrease within- and between-person momentary NA, social support could mitigate stress and improve psychological well-being among police officers [40, 41]. Therefore, police organizations should encourage police officers to engage in social relationships with family and friends, through guaranteed off-duty and rest time.

### Limitations

This study used a mobile application that is only available for Android smartphones, and the lack of accessibility created a technical issue that we will remedy in future studies. Moreover, due to missing data, survey completion rates may have impacted the results of the EMA study. Therefore, the interpretation of the results could be limited. The completion rate for this study was 64.7%, and a systematic review of EMA methodology reported ranges from 44 to 96% [42]. A previous study reported that a study’s completion rate may be influenced by physiological factors such chronic pain, as well as by psychological aspects [21, 43, 44]. Although there is no gold standard for completion rates, it is important to ensure the generalizability of study results. Therefore, researchers conducting an EMA study should consider participants’ possible physiological and psychological difficulties before and during the study.

In this study, the median response latency was 38 min. A systematic review of diet and physical activity in youth reported response latency longer than 15 min in 0 to 40% of studies using electronic devices, compared to 71.7 to 74.1% for paper-and-pencil methods [42]. A study reported an average response latency of 29 min [45], and another reported 19 min [46]. The participants of those studies were youth (e.g., high school or undergraduate students) who probably responded faster to an EMA than our participants. In the present study, we attempted not to impose a time limit in order to reduce the burden of the survey on participants and to increase response rates. Although this was to account for the possibility that participants could not respond immediately, due to being occupied with other tasks or sleeping after work, momentary stress factors and affect may have been underestimated. In future research, strategies to reduce response latency should be used when measuring time-sensitive variables. Additionally, other factors that were not included in this study—such as various work-related events, relationships, resilience, and fatigue—may have influenced the results.

As the police officers who participated in this study worked in different departments, their work characteristics are not generalizable to all police officers. Further, generalizability of the results is also limited, as the participants did not work in departments with a relatively high demand for security. We did not investigate duty schedules, date information (weekday or weekend), or other potential factors, such as fatigue, which can also influence affect and stress.

Some questionnaire items may have ambiguous meanings. For example, participants might have interpreted an item as “I feel good because I did a lot of work,” rather than “I am tired because I did a lot of work.” Furthermore, “I try to maintain a good relationship with others” could be interpreted positively as “challenging” rather than “stressful.” Thus, momentary PA might be positively responsive to momentary stress.

Lastly, we acknowledge that multiple testing in statistical analysis may increase the risk of type 1 errors (false positives); however, the risk was low in this study, considering that our statistically significant effects were consistent across the entire study group [47]. Despite these limitations, as police officers’ momentary stress and affect were measured in real-time, it can be said that their momentary stress and affect were sufficiently reflected in this study, as recall bias was low.

## Conclusion

Our findings provided the first real-time evidence that identified the momentary stress factors that influence momentary affect among police officers. Our results also showed that momentary PA and NA were influenced by stress and contextual factors at the within- and between-person levels. The findings of this study will be useful for understanding police officers’ stress and affect, and in generating new approaches to improving their psychological health.

## Supplementary information

**Additional file 1: Table S1.** TICS questionnaire.

## Data Availability

The datasets are available from the corresponding author on reasonable request.

## Abbreviations

EMA: Ecological momentary assessment
PA: Positive affect
NA: Negative affect
