# Supplementary material for: Evaluating real-time momentary stress and affect in police officers using a smartphone application
Source: BMC Public Health. 2020 Jul 23;20:1154. doi: 10.1186/s12889-020-09225-z (PMC7376902; doi:10.1186/s12889-020-09225-z)
Supplement: Supplementary file 1 — Additional file 1: Table S1. TICS questionnaire. [file 12889_2020_9225_MOESM1_ESM.docx]

Supplementary 1

Table S1. TICS questionnaire

| Construct | Items |
| --- | --- |
| 1. Work overload | I did a lot of work |
| 2. Social overload | I dealt a lot with other people’s matters |
| 3. Excessive demands at work | I performed some of my tasks inadequately |
| 4. Lack of social recognition | Others undervalued my work |
| 5. Work discontent | I felt discontented with the type of work I am doing |
| 6. Social tension | I had a disagreement with someone |
| 7. Pressure to perform | I performed tasks that allowed no mistakes |
| 8. Social isolation | It was important to ensure good relations with another person |
